# Does C-reactive protein predict time to recovery and benefit from oseltamivir treatment in primary care patients with influenza-like illness? A randomized controlled trial secondary analysis

**DOI:** 10.1080/02813432.2021.2006482

**Published:** 2021-12-01

**Authors:** Nicolay Jonassen Harbin, Karin Rystedt, Morten Lindbaek, Ruta Radzeviciene, Johan Westin, Ronny Gunnarsson, Christopher C. Butler, Alike W. van der Velden, Theo J. Verheij, Pär-Daniel Sundvall

**Affiliations:** a Antibiotic Center for Primary Care, Department of General Practice, Institute of Health and Society, University of Oslo, Oslo, Norway; b Research, Education, Development and Innovation, Primary Health Care, Region Västra Götaland, Sweden; c General Practice/Family Medicine, School of Public Health and Community Medicine, Institute of Medicine, Sahlgrenska Academy, University of Gothenburg, Gothenburg, Sweden; d Centre for Antibiotic Resistance Research (CARe) at University of Gothenburg, Gothenburg, Sweden; e Region Västra Götaland, Närhälsan Stenstorp vårdcentral, Stenstorp, Sweden; f Mano Seimos gydytojas, Klaipeda, Lithuania; g Department of Infectious Diseases, Institute of Biomedicine, University of Gothenburg, Gothenburg, Sweden; h Department of Primary Care Health Services, University of Oxford, Radcliffe Observatory Quarter, Oxford, UK; i Julius Center for Health Sciences and Primary Care, University Medical Center Utrecht, Utrecht, the Netherlands

**Keywords:** C-reactive protein, influenza-like illness, recovery time, antiviral treatment, respiratory tract infections, primary health care

## Abstract

**Objective:**

Recovery time and treatment effect of oseltamivir in influenza-like illness (ILI) differs between patient groups. A point-of-care test to better predict ILI duration and identify patients who are most likely to benefit from oseltamivir treatment would aid prescribing decisions in primary care. This study aimed to investigate whether a C-reactive protein (CRP) concentration of ≥30 mg/L can predict (1) ILI disease duration, and (2) which patients are most likely to benefit from oseltamivir treatment.

**Design:**

Secondary analysis of randomized controlled trial data.

**Setting:**

Primary care in Lithuania, Sweden and Norway during three consecutive influenza seasons 2016–2018.

**Subjects:**

A total of 277 ILI patients aged one year or older and symptom duration of ≤72 h.

**Main outcome measures:**

Capillary blood CRP concentration at baseline, and ILI recovery time defined as having ‘returned to usual daily activity’ with residual symptoms minimally interfering.

**Results:**

At baseline, 20% (55/277) had CRP concentrations ≥30mg/L (range 0–210). CRP concentration ≥30 mg/L was not associated with recovery time (adjusted hazards ratio (HR) 0.80: 95% CI 0.50–1.3; *p* = 0.33). Interaction analysis of CRP concentration ≥30 mg/L and oseltamivir treatment did not identify which patients benefit more from oseltamivir treatment (adjusted HR 0.69: 95% CI 0.37–1.3; *p* = 0.23).

**Conclusion:**

There was no association between CRP concentration of ≥30 mg/L and recovery time from ILI. Furthermore, CRP could not predict which ILI patients benefit more from oseltamivir treatment. Hence, we do not recommend CRP testing for predicting ILI recovery time or identifying patients who will receive particular benefit from oseltamivir treatment.

**Key Points**
Predicting disease course of influenza-like illness (ILI), and identifying which patients benefit from oseltamivir treatment is a challenge for physicians.
• There was no association between CRP concentration at baseline and recovery time in patients consulting with ILI in primary care.
• There was no association between CRP concentration at baseline and benefit from oseltamivir treatment.
• We, therefore, do not recommend CRP testing for predicting recovery time or in decision-making concerning oseltamivir prescribing in ILI patients.

## Introduction

Influenza viruses account for considerable morbidity and mortality [1]. Each year, approximately 20% of children and 5% of adults worldwide get infected during annual winter epidemics caused by Influenza A and B [2]. Together with other viruses, Influenza A and B cause influenza-like illness (ILI) [3], which is defined by the World Health Organization as an acute respiratory illness with a temperature of ≥38 °C and cough, with onset within the past 10 days [4].

ILI symptoms usually last between one to two weeks [5], and the duration is influenced by previous immunisation, viral factors, patients’ age, comorbidity and symptom severity [2,6].

Although European Guidelines recommend antiviral treatment for patients presenting with suspected or confirmed influenza, this group of drugs is not commonly prescribed in most European countries [7]. Two meta-analyses have found that oseltamivir improves time to first alleviation of symptoms by 16.8 h [8], and median time to alleviation of symptoms by 17.8 h [9]. Our study is part of a recent European multicentre study on treatment of ILI in primary care: We found an estimated mean benefit in time to recovery of 1.02 days overall by adding oseltamivir to usual care as compared with usual care alone. An increasing benefit up to two–three days sooner recovery was seen in older, sicker patients with longer previous symptom duration and comorbidities [6]. The large variation in treatment effect between patient groups poses the question if there might be additional predictors which more precisely identify which patients could benefit more from oseltamivir treatment.

In daily practice, it can be challenging to predict recovery in individuals presenting with ILI, and which patients with suspected or confirmed influenza benefit the most from antiviral medication. Hence, a point of care test (POCT) biomarker able to predict outcomes could have clinical utility. C-reactive protein (CRP), a low-cost POCT is commonly used in Scandinavian primary care in managing respiratory tract infections [10–14].

CRP is a non-specific acute-phase reactant produced in the liver in response to inflammation and tissue injury [15]. The concentration of CRP correlates with systemic inflammation [16]. A common finding in viral respiratory tract infections is moderately increased concentrations of CRP (10–60 mg/L) [17]. In primary care, CRP has not associated with influenza A infections in patients with ILI [18].

CRP concentration measured in an early stage in hospitalized patients presenting with influenza A infection has been found to predict the severity and the outcome of disease [19,20]. To our knowledge, only one previous study has investigated CRP as a predictor for time until recovery from influenza A [21], which was done in an emergency department. We have not identified previous studies investigating CRP as a predictor for antiviral treatment effect.

The aims of this study were to investigate whether the CRP concentration of ≥ 30mg/L measured on the day of first consultation in primary care for ILI can predict (1) disease duration until recovery, and (2) which patients benefit more from treatment with oseltamivir.

## Methods

Patients aged one year and older seeking primary care for ILI were eligible for inclusion. This was a secondary analysis of data from ALIC^4^E, an open-label multicentre RCT on the effectiveness of oseltamivir treatment for patients with ILI in primary care [6]. ALIC^4^E recruited 3266 participants in 15 European countries. This secondary analysis used the 277 patients recruited from 30 primary care practices in Sweden, Lithuania and Norway where CRP was measured upon inclusion. This was the same subset as used in a study investigating the association of CRP with influenza A or B infection in patients with ILI [18].

### Inclusion and procedures

Recruitment of patients took place during three seasonal influenza epidemics between 27 January 2016, and 4 April 2018. Patients ≥1 year of age presenting to primary care with ILI, able to comply with study requirements and who agreed to take an antiviral agent according to randomization were eligible for inclusion. ILI was defined as a sudden onset of self-reported fever, with ≥1 respiratory symptom (cough, sore throat, running or congested nose) and ≥1 systemic symptom (headache, muscle ache, sweats or chills, or tiredness), with symptom duration of ≤72 h at time of inclusion. Exclusion criteria are described earlier, with the main exclusion criterion being the need for acute hospital admission [6]. Pregnant, lactating or breastfeeding patients were excluded in Lithuania, due to country-specific legislation. A baseline case-report form was completed at inclusion covering symptom duration before recruitment, relevant comorbidity (e.g. asthma, COPD, diabetes, etc.) and physician rated ILI severity (mild, moderate or severe), among other factors. Patients completed a symptom diary for 14 days, self-assessing fever, respiratory symptoms (cough, sore throat, runny/congested nose, shortness of breath), abdominal symptoms (diarrhoea, nausea/vomiting and abdominal pain) and systemic symptoms (headache, sweats/chills, muscle ache, low/energy/tiredness, feeling generally unwell and dizziness) with the scoring possibilities of ‘no’, ‘minor’, ‘moderate’ and ‘major’ problem. To support diary and study completion, as well as obtaining minimal data on recovery, patients were followed up *via* telephone calls after days 2–4, 14–28 days and 28 days.

All participants received verbal and written information about the study prior to inclusion, and written consent was provided before participation. All study procedures were performed according to the ethical principles of the Declaration of Helsinki.

### Measurements

The primary outcome was days until recovery, defined as having ‘returned to usual daily activity’, with ‘fever’, ‘muscle ache’ and ‘headache’ rated as a ‘minor’ or ‘no’ problem. Capillary blood samples were taken at inclusion for the measurement of CRP concentration. CRP analyses were performed locally with the available POCT devices routinely used by the participating practices. Devices used in Sweden and Norway only measured concentrations ≥5 mg/L, while the devices used in Lithuania measured concentrations ≥0 mg/L. For detection of influenza A and B a nasal and an oropharyngeal swab (COPAN®) were used in patients aged <16 years and a nasopharyngeal swab (COPAN®) was used in patients ≥16 years of age. The procedures for swab analyses have been described previously [6].

### Statistical analysis

A Kaplan–Meier survival curve was produced for both treatment arms as one group to avoid result duplication from the main RCT. Hazard ratios (HR) for baseline capillary blood CRP to influence days until recovery was the main outcome of interest and estimated with multivariable Cox regression. Time to recovery was the dependent variable and independent variables were CRP concentration ≥30 mg/L, oseltamivir treatment, interaction analysis between CRP and oseltamivir treatment, increasing age in decades, male gender, Influenza A, Influenza B, duration of ILI symptoms, severity of ILI and chronic respiratory conditions. The analysis was performed on all patients with follow-up data. Missing data were not imputed. The rational for using a cut-off for CRP of 30 mg/L has been described previously [18].

The level of significance was set to 0.05 and IBM SPSS version 25 was used.

## Results

During three consecutive influenza seasons, 281 patients in total were recruited, with CRP results available for 277 patients. Patients registered with a chronic respiratory condition, for example, asthma or chronic obstructive pulmonary disease, constituted 7.6% (21/277) of all patients. Of participants, 145 (52%) were randomized to oseltamivir plus usual care, while the remaining 132 (48%) were randomized to usual care alone. Additional patient characteristics and influenza aetiology results are described by Rystedt et al. [18].

### CRP concentrations, symptom severity scoring, treatment effect

**Figure 1.**
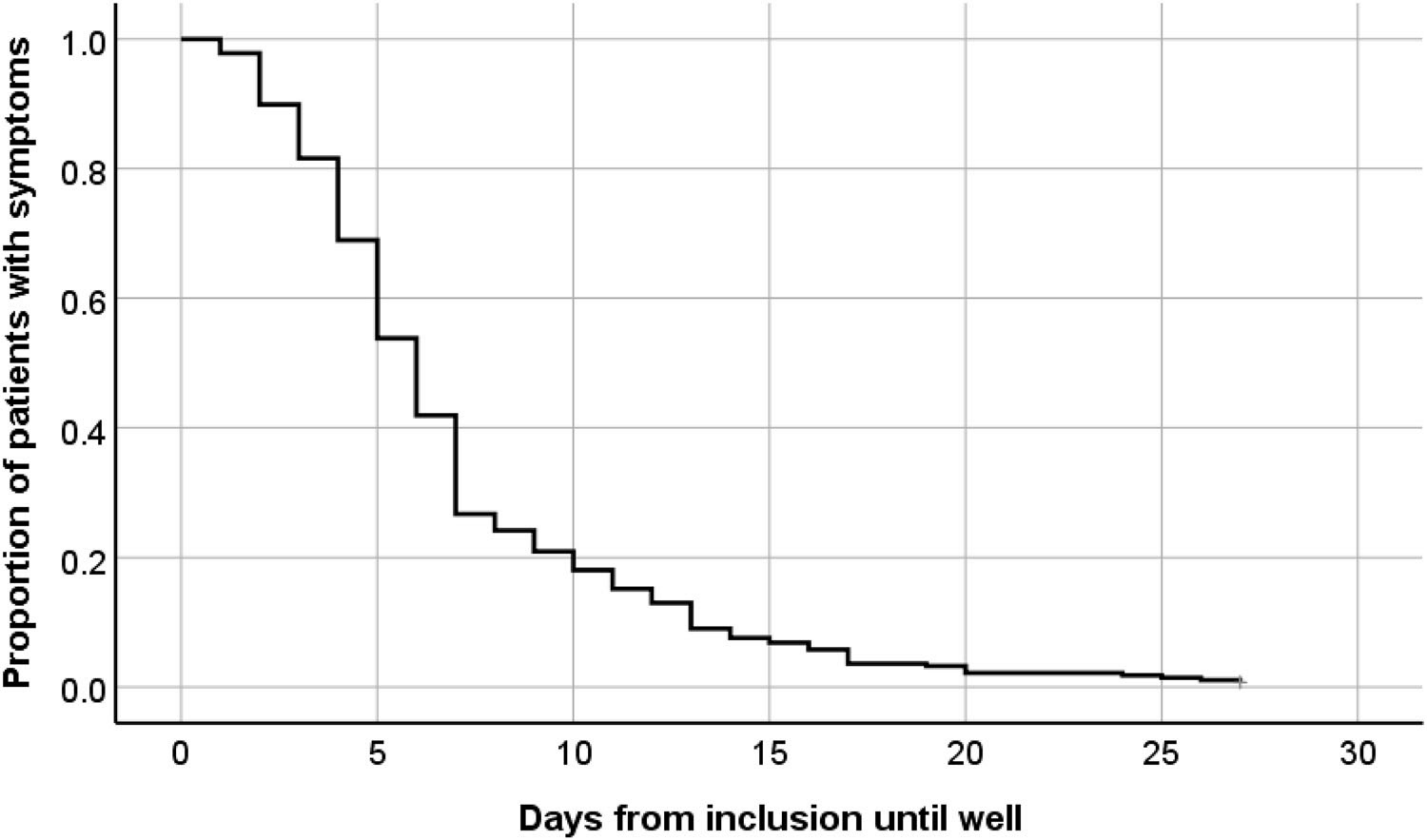
Number of days to recovery, defined as return to usual activities, with fever, headache, and muscle ache minor or absent.

At baseline 20% (55/277) of participants had a CRP concentration ≥30mg/L (range 0-210) (Table 1). Physician-assessed scoring of ILI severity at inclusion showed 20% of patients (57/277) rated as mild, 65% (179/277) as moderate and 15% (41/277) as severe (Table 1). Median time until recovery for patients in both arms together was 6 days, while approximately 80% had recovered within 10 days (Figure 1). Mean days until recovery was 7.6 days (SD 5.0) in the usual care group as compared to 6.6 days (SD 4.7) in the oseltamivir plus usual care group (Table 1).

**Table 1.** Characteristics of participants.

	Usual care	Oseltamivir plus usual care
CRP≥ 30 mg/L	19% (25/132)	21% (30/145)
Severity of influenza-like illness		
Mild	24% (31/132)	18% (26/145)
Moderate	64% (84/132)	66% (95/145)
Severe	13% (17/132)	17% (24/145)
Presence of influenza		
Influenza A	42% (56/132)	45% (65/145)
Influenza B	19% (25/132)	23% (33/145)
Not influenza A or B	39% (51/132)	32% (47/145)
Mean age in years (SD) (*n* = 277)	31 (19)	33 (19)
Female gender	57% (75/132)	58% (84/145)
Chronic respiratory condition^a^	6.8% (9/132)	8.3% (12/145)
Country		
Lithuania	58% (76/132)	54% (78/145)
Sweden	23% (30/132)	27% (39/145)
Norway	20% (26/132)	19% (28/145)
Days until recovery (*n* = 273)		
Median days (IQ-range)	6.0 (5.0-9.5)	5.0 (3.0-7.5)
Mean days (SD)	7.6 (5.0)	6.6 (4.7)

^a^For example, asthma or chronic obstructive pulmonary disease.

### Factors associated with time to recovery from influenza-like illness

Time to recovery (return to usual daily activities with residual ILI symptoms minimally interfering) could not be predicted based on a CRP concentration with a cut-off of ≥30 mg/L (adjusted HR of 0.80; 95% CI 0.50–1.3; *p* = 0.33, Table 2). Oseltamivir treatment was associated with a quicker recovery time (adjusted HR of 1.5; 95% CI 1.2–2.0; *p* = 0.0021). Interaction analysis between CRP with a cut-off of ≥30 mg/L and oseltamivir treatment could not identify which patients benefit more from oseltamivir treatment (adjusted HR of 0.69; 95% CI 0.37–1.3; *p* = 0.23, Table 2).

**Table 2.** Factors associated with time to recovery^a^ from influenza-like illness (ILI) (*n* = 277).

	Hazards ratio^b^ (CI 95%)	*p*-value
CRP ≥ 30 mg/L	0.80 (0.50–1.3)	0.33
Oseltamivir treatment	1.5 (1.2–2.0)	0.0021
Interaction between CRP and treatment^c^	0.69 (0.37–1.3)	0.23
Increasing age (decades)	0.85 (0.79–0.91)	<0.001
Male gender	1.0 (0.79–1.3)	0.86
Influenza A	0.99 (0.74–1.3)	0.93
Influenza B	0.82 (0.58–1.2)	0.25
Duration of ILI symptoms	0.98 (0.84–1.1)	0.82
Severity of influenza-like illness^d^	0.87 (0.69–1.1)	0.21
Chronic respiratory condition^e^	0.62 (0.39–0.99)	0.045

^a^Defined as return to usual daily activities, with fever, headache, and muscle ache minor or absent.

^b^HR >1.0 indicates quicker recovery, 273 included in the analysis.

^c^Interaction between CRP with a cut-off of ≥30 mg/L and oseltamivir treatment for influenza-like illness.

^d^HR for an increase of one step in severity coded as mild, moderate or severe.

^e^For example, asthma or chronic obstructive pulmonary disease.

Increasing age in decades and having a chronic respiratory condition were associated with longer recovery times with adjusted HRs of 0.85 (95% CI 0.79–0.91; *P* ≤ 0.001) and 0.62 (95% CI 0.39–0.99; *p* = 0.045) respectively (Table 2). We did not find significant associations between recovery time and severity of ILI or influenza aetiology.

## Discussion

We did not find an association between C-reactive protein concentration of ≥30 mg/L at first consultation for ILI in primary care and time to recovery. Neither did we find any significant interaction between CRP and oseltamivir treatment for recovery time. Overall, median time from first visit to recovery was six days, while increasing age and presence of a chronic respiratory condition were associated with prolonged recovery time.

### Strength and limitations

The randomized, prospective recruitment process of the study with a symptom-based inclusion approach is a main strength of this study. The trial`s pragmatic design additionally reflects everyday primary care practice with patients seeking care, regardless of symptom severity, comorbidities and age, and with a broad variety of ILI symptoms. We obtained CRP concentrations after the inclusion of participants, so that the CRP concentration did not influence the physicians’ choice to include the patient or the randomization process. Finally, by the open-label design of the main study, it is likely that we describe real-world effects seen in primary care. We therefore believe the results are generalizable and applicable to primary care in other countries. The low number of older patients (13/277) and the somewhat low number of patients having severe ILI (41/277) as assessed by the recruiting physician are considered the main limitations of the study. Furthermore, recruitment of patients took place over a three-year period and it is likely that to a certain extent eligible patients have not been included due to busyness of the recruiting physician and time-consuming inclusion procedures. In the assessment of this, we nevertheless consider it unlikely to have caused a selection bias.

### Factors associated with recovery time from ILI

Participants in our study had a median recovery time of approximately one day sooner as compared to a previous Norwegian primary care study conducted during the peak of the H1N1 pandemic in 2009, reporting an overall median recovery time of seven days [5]. Regarding possible differences in definition of recovery and the different proportions of patients prescribed oseltamivir treatment in the two studies (39% vs. 52%), this comparison should be interpreted with caution. Our data also showed that adding oseltamivir to usual care reduced mean recovery time of ILI by approximately one day, which is consistent with the larger dataset in the main study [6]. We did not find a correlation between time to recovery and CRP concentration. This is contrary to the findings of Haran et al. which, to our knowledge, is the only other study investigating CRP as a predictor for recovery time in influenza patients. They found that patients with laboratory confirmed Influenza A with CRP concentrations over 25 mg/L had an average symptom duration of 13.4 days (95% CI 7.6–19.3) as compared to 8.2 days (95% CI 5.3–11.1) in the less than 25 mg/L group [21]. Various issues could explain this discordant finding, a difference in the sizes of study population (24 patients vs. 277), in aetiology (laboratory confirmed influenza A vs. a wider aetiology of ILI), and in setting and severity (inclusion at an emergency department vs. primary care). Older age and a chronic respiratory condition are well-known risk factors for severe disease or complications from influenza infections [3]. We found an association between prolonged recovery time and the above-mentioned risk factors, in line with the findings of the main study and a previous meta-analysis [6,9]. The Kaplan–Meier survival curve (Figure 1) was produced for all participants regardless of CRP concentrations, as CRP concentrations ≥30 mg/L was not associated with recovery time.

The second aim of the study was to investigate if CRP could predict which patients benefit more from oseltamivir treatment. Our interaction analysis did not reveal that patients with a CRP ≥30 mg/L had a better effect of oseltamivir treatment.

## Conclusions

CRP concentration ≥30 mg/L did not predict time to recovery, neither did this binary CRP cut-point predict which patients received increased benefit from oseltamivir treatment. We therefore do not recommend the use of CRP for predicting recovery time of ILI, or as a tool for use in addition to established influenza risk factors for evaluating which patients should be prescribed oseltamivir treatment or not for ILI.
